# Trends and uptake of new formulations of controlled‐release oxycodone in Canada

**DOI:** 10.1002/pds.4390

**Published:** 2018-01-23

**Authors:** Tara Gomes, Sonia Jain, J. Michael Paterson, Ingrid Sketris, Patricia Caetano, David Henry, the Canadian Network for Observational Drug Effect Studies (CNODES) Investigators

**Affiliations:** ^1^ Li Ka Shing Knowledge Institute of St. Michael's Hospital Toronto Ontario Canada; ^2^ Institute for Clinical Evaluative Sciences Toronto Ontario Canada; ^3^ Institute of Health Policy, Management, and Evaluation Toronto Ontario Canada; ^4^ Leslie Dan Faculty of Pharmacy University of Toronto Toronto Ontario Canada; ^5^ Faculty of Kinesiology and Physical Education University of Toronto Toronto Ontario Canada; ^6^ Department of Family Medicine McMaster University Hamilton Ontario Canada; ^7^ College of Pharmacy Dalhouse University Halifax Regional Municipality Nova Scotia Canada; ^8^ Faculty of Health Sciences University of Manitoba Manitoba Canada; ^9^ Provincial Drug Programs Government of Manitoba Manitoba Canada; ^10^ Centre for Research in Evidence‐based Practice Bond University Gold Coast Australia

**Keywords:** drug policy, opioid analgesics, pharmacoepidemiology

## Abstract

**Purpose:**

This study investigated the impact of changing availability of tamper‐deterrent and non‐tamper‐deterrent oxycodone on prescribing patterns of controlled‐release oxycodone across Canada.

**Methods:**

We conducted a population‐based, serial cross‐sectional study of controlled‐release oxycodone dispensing from community pharmacies across Canada between October 2007 and April 2016. We calculated rates of dispensing (tablets per 100 population) and reported the relative market share of generic non‐tamper‐deterrent controlled‐release oxycodone. All analyses were reported nationally and stratified by province.

**Results:**

After the introduction of a tamper‐deterrent formulation, the national rate of controlled‐release oxycodone dispensing fell by 44.6% (from 26.4 to 14.6 tablets per 100 population from February 2012 to April 2016). Between December 2012 and July 2013, there was moderate uptake of generic non‐tamper‐deterrent controlled‐release oxycodone (968 452 tablets; 16.0% in July 2013), which appeared to have little impact on the overall rate of controlled‐release oxycodone dispensing in Canada. However, the uptake of generic non‐tamper‐deterrent oxycodone varied considerably by province. By April 2016, 55.0% of all controlled‐release oxycodone tablets dispensed in Quebec were for the generic formulation. Elsewhere in Canada, this prevalence was less than 30%, ranging between 1.6% (Prince Edward Island) and 26.9% (British Columbia) at the end of our study period.

**Conclusions:**

The changing availability of tamper‐deterrent and non‐tamper‐deterrent formulations of controlled‐release oxycodone in Canada has had variable influence on the rate of use of these products across Canada. Future research should explore whether the availability of generic controlled‐release oxycodone has led to measurable changes in the safety of oxycodone use in Canada.

## 1 INTRODUCTION

Opioids are a class of drugs that have been traditionally used to treat acute and chronic pain. Despite fairly stable prescribing patterns in the 1980s and early 1990s, opioid prescribing rose dramatically in Canada following the approval of OxyContin,^1^ a controlled‐release formulation of oxycodone in 1996. This was largely attributed to an intense marketing campaign by OxyContin's manufacturer, which promoted the use of controlled‐release opioids for the treatment of chronic noncancer pain, with claims of minimal risk of addiction.^2^, ^3^ Over the subsequent decade, the addiction potential of controlled‐release opioid formulations was realized, evidenced by an epidemic of prescription opioid misuse and related overdose deaths across North America.^1^, ^4^, ^5^, ^6^

Since this time, policies have been introduced across Canada to address the rising use of oxycodone and its potential for misuse. These have included restricted access on some provincial drug programs and the introduction of safety warnings on product monographs.^7^, ^8^ In February 2012, the manufacturer of OxyContin introduced a tamper‐deterrent formulation of controlled‐release oxycodone (OxyNeo) across Canada and ceased production of their original product.^9^ Although this new formulation does not protect against developing an opioid addiction or accidentally overdosing when ingesting the drug orally, it was designed to be more difficult to crush, chew, or dissolve thus introducing barriers to its misuse.^9^ The introduction of this product coincided with restricted listing status of controlled‐release oxycodone products on the public formularies in several provinces (Appendix S1).^7^ In November 2012, the oxycodone prescribing landscape shifted again when the patent for OxyContin expired, and Health Canada approved generic forms of controlled‐release oxycodone without tamper‐deterrent properties.^10^ In Australia, a similar decision to Canada was implemented in 2014. A published conference paper reported that the introduction of the tamper‐deterrent form of oxycodone led to some switching to other opioids, but the authors found no discernible additional impact of the introduction of the generic products on opioid‐switching patterns.^11^ In contrast, in the United States, the Food and Drug Administration announced that it would not approve any forms of generic OxyContin because of concerns that the risks outweighed the benefits of this non‐tamper‐deterrent form of controlled‐release oxycodone.^12^ The approval of these drugs in Canada led to debate regarding the safety of reintroducing non‐tamper‐deterrent oxycodone to the market and decisions on the part of many Canadian provincial drug programs against listing the generic formulations on their formularies.^13^, ^14^, ^15^ The generic long‐acting forms of oxycodone are marketed in all Canadian provinces and can be prescribed by a registered medical practitioner (and in some provinces, a nurse practitioner) to any patient who is believed to need a controlled drug for pain relief. The prescription may be subject to special monitoring (eg, systems to avoid inappropriate “double doctoring,” which vary by province) and the patient, and/or their insurer, is responsible for covering the dispensed drug price. For example, the cost of 100 tablets of 20‐mg generic long‐acting oxycodone is approximately $66 plus a pharmacist's professional fee. In Canada, the generic drug is covered by only 2 provincial public drug plans, Quebec and Nova Scotia (see Appendix S1), which have different levels of premiums, co‐payment, and deductibles. In Quebec, patients can request a generic drug formulation from their pharmacist at time of dispensing unless their doctor has specified “no substitution” on their OxyNeo prescription, which would prevent the pharmacist from dispensing the generic non‐tamper‐deterrent formulation. In contrast, in Nova Scotia, pharmacists cannot substitute OxyNeo with generic controlled‐release oxycodone.

Despite these changes, and the associated controversies, little is known about the uptake of OxyNeo and generic controlled‐release oxycodone across the country. As policy‐makers and drug regulators debate the role that tamper‐deterrent opioid formulations can have in addressing opioid misuse and addiction, more information is needed to understand shifts in prescribing patterns when these drugs are reformulated. This study sought to determine the impact of changing availability of controlled‐release oxycodone formulations on rates of dispensing across Canada.

2

**KEY POINTS**

- The introduction of a tamper‐deterrent formulation was associated with a 44.6% reduction in overall dispensing of controlled release oxycodone.
- There was moderate uptake of generic, non‐tamper‐deterrent oxycodone; however, this did not lead to an expansion in the overall prescribing rate of controlled‐release oxycodone.
- By 2016, 1 in 5 tablets for controlled‐release oxycodone that were dispensed in Canada were for a generic, non‐tamper‐deterrent form.
- Uptake of generic non‐tamper‐deterrent oxycodone was highest in Quebec, where more than half of all controlled‐release oxycodone is in this form.

## 3 METHODS

We conducted a population‐based, serial cross‐sectional study of controlled‐release oxycodone dispensing from community pharmacies across Canada between October 2007 and April 2016. We used the QuintilesIMS Compuscript database to quantify prescription volumes for all long‐acting single‐agent oxycodone products dispensed over the study period, including those covered by provincial public insurance plans, private insurance companies and those purchased out of pocket. This database provides projections for prescription quantities at the provincial and national level based on data captured from a representative sample of approximately 6000 community pharmacies across the country. The geographic location of pharmacies, the distance between pharmacies and pharmacy size are all incorporated into projections, which are conducted by QuintilesIMS at the level of drug identification number. The quality of these data is continuously monitored and verified by QuintilesIMS, and the Compuscript database is regularly used for research purposes.^1^, ^16^, ^17^ We calculated the national and provincial monthly rate of controlled‐release oxycodone dispensing as the total number of tablets dispensed per 1000 population using provincial population estimates from the 2006 Statistics Canada census as our denominator. The national market share of each formulation of controlled‐release oxycodone was calculated in each month over the study period as the number of controlled‐release oxycodone tablets that were dispensed for each formulation (OxyContin, OxyNeo, and generic controlled‐release oxycodone). Finally, we estimated the monthly proportion of controlled‐release oxycodone dispensing that was for the generic formulations between December 2012 (when generic formulations were first marketed) and the end of the study period. This was calculated as the total number of generic controlled‐release oxycodone tablets dispensed divided by the total number of controlled‐release oxycodone tablets dispensed nationally and stratified by province.

## 4 RESULTS

**Figure 1:**
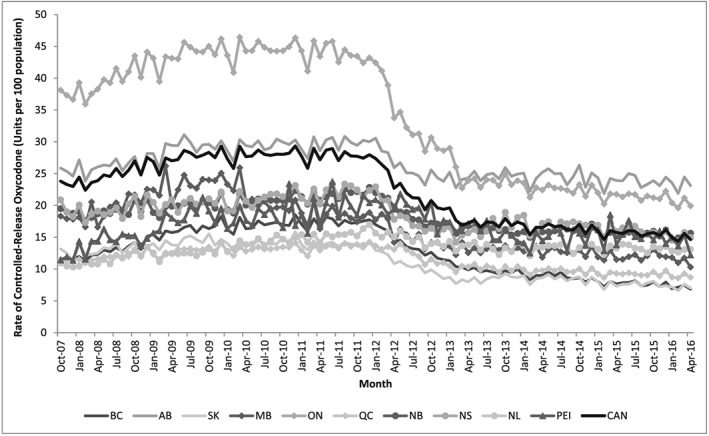
Rate of controlled‐release oxycodone dispensing in Canada, stratified by province. October 2007 to April 2016. Legend: monthly rates of controlled‐release oxycodone tablet dispensing per 100 population are reported nationally and by province. Provinces listed with approximate population as name (average population in millions over the study period). BC: British Columbia (4.5 million); AB: Alberta (3.8 million); SK: Saskatchewan (1.1 million); MB: Manitoba (1.2 million); ON: Ontario (13.2 million); QC: Quebec (8.0 million); NB: New Brunswick (0.8 million); NS: Nova Scotia (0.5 million); NL: Newfoundland and Labrador (0.5 million); PEI: Prince Edward Island (0.1 million); CAN: Canada (34.2 million)

A total of 780.2 million tablets for controlled‐release oxycodone were dispensed across Canada over the study period, the majority of which (457.2 million; 58.6%) were dispensed in Ontario. In October 2007, the rate of controlled‐release oxycodone dispensing ranged from a low of 11.0 tablets per 100 population in Newfoundland and Labrador to 38.1 tablets per 100 population in Ontario (Figure 1). On a national level, the rate of controlled‐release oxycodone dispensing was initially relatively consistent, ranging between 22.4 and 29.3 tablets per 100 population between October 2007 and February 2012 (Figure 1). Upon the introduction of a tamper‐deterrent formulation at that time, the rate of controlled‐release oxycodone dispensing fell by 44.6% from 26.4 tablets per 100 population in February 2012 to 14.6 tablets per 100 population in April 2016. This fall was seen in most provinces but was variable, ranging from 12.6% in Newfoundland and Labrador (from 15.0 to 13.1 tablets per 100 population) to 58.5% in British Columbia (from 16.5 to 6.8 tablets per 100 population). There was no apparent rebound in dispensing after the introduction of generic forms in late 2012 (Figure 1).

**Figure 2:**
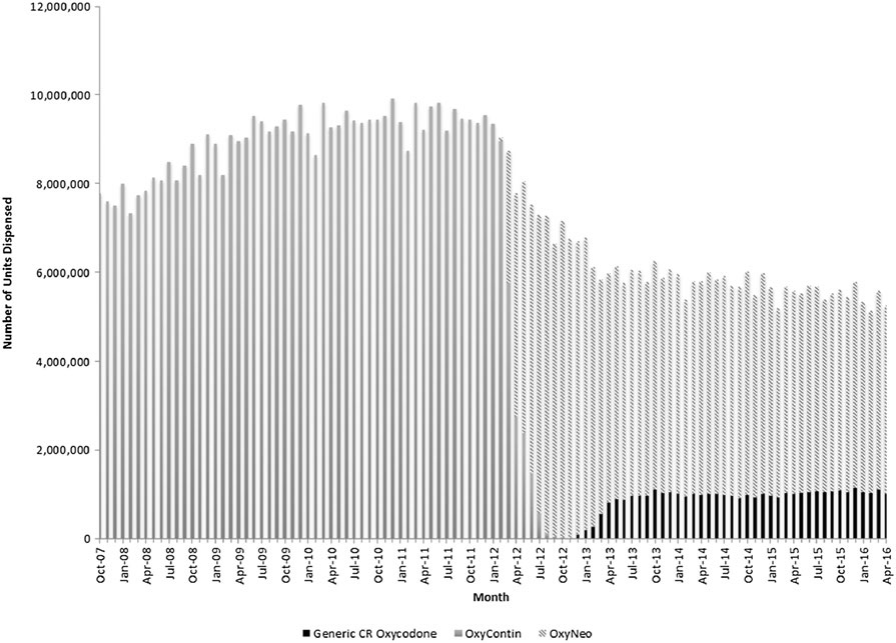
Canadian market share of controlled‐release oxycodone products. October 2007 to April 2016. Legend: total number of controlled‐release oxycodone tablets dispensed nationally, stratified by formulation. Formulations are as follows: OxyContin = brand name, non‐tamper‐deterrent controlled‐release oxycodone; OxyNeo = brand name, tamper‐deterrent controlled‐release oxycodone; generic CR oxycodone = generic, non‐tamper‐deterrent controlled‐release oxycodone

**Figure 3:**
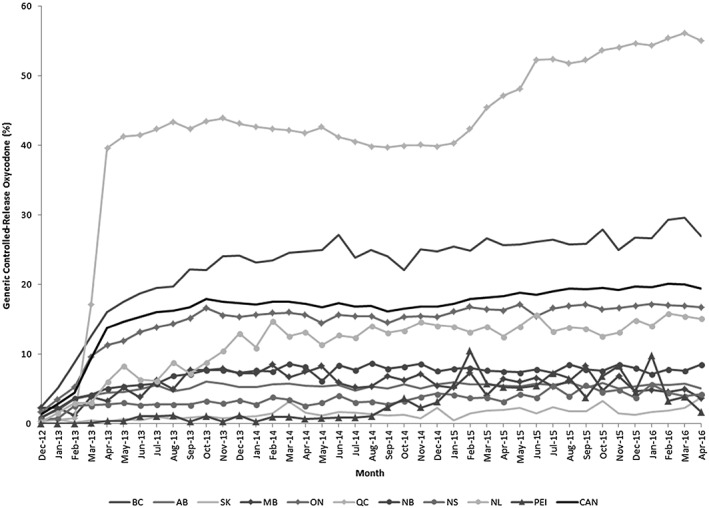
Prevalence of generic controlled‐release oxycodone by Canadian province. December 2012 to April 2016. Legend: monthly proportion of all controlled‐release oxycodone that is a generic formulation, reported as a percentage nationally and by province. Provinces listed with approximate population as name (average population in millions over the study period). BC: British Columbia (4.5 million); AB: Alberta (3.8 million); SK: Saskatchewan (1.1 million); MB: Manitoba (1.2 million); ON: Ontario (13.2 million); QC: Quebec (8.0 million); NB: New Brunswick (0.8 million); NS: Nova Scotia (0.5 million); NL: Newfoundland and Labrador (0.5 million); PEI: Prince Edward Island (0.1 million); CAN: Canada (34.2 million)

The national market shares of the different controlled‐release oxycodone products changed considerably over our study period. Upon its introduction in February 2012, the tamper‐deterrent formulation (OxyNeo) quickly replaced OxyContin, which was voluntarily withdrawn from the market by its manufacturer^9^ (Figure 2). Following the approval of generic non‐tamper‐deterrent forms of controlled‐release oxycodone in December 2012, there was a modest uptake of these tablets such that 968 452 tablets (16.0% of all controlled‐release oxycodone) were dispensed in Canada by July 2013 (Figure 2). Following this initial increase, the monthly volume of generic forms of controlled‐release oxycodone remained stable, ranging from 916 055 to 1 139 643 tablets (16.0% to 20.0% of all controlled‐release oxycodone), over the remainder of the study period (Figures 2 and 3).

The uptake of generic controlled‐release oxycodone varied considerably across Canada, with a range of 1.6% to 55.0% across the Canadian provinces in April 2016 (Figure 3). In Quebec, there was rapid uptake of this formulation between February and April 2013, after which the prevalence of generic dispensing remained relatively stable until another rise occurred in early 2015. Overall, the prevalence of generic dispensing reached 39.6% in April 2013, and by April 2016, 55.0% of all controlled‐release oxycodone tablets dispensed in Quebec were for the generic formulation. We also observed considerable uptake of generic controlled‐release oxycodone in British Columbia (26.9% prevalence in April 2016), Ontario (16.7% prevalence in April 2016), and Newfoundland and Labrador (15.0% prevalence in April 2016). In all other provinces, the prevalence of generic controlled‐release oxycodone dispensing remained relatively low, ranging between 1.6% (Prince Edward Island) and 8.5% (New Brunswick) at the end of our study period (Figure 3).

## 5 DISCUSSION

In this repeated cross‐sectional study of controlled‐release oxycodone dispensing patterns across Canada, we found reductions in the rate of dispensing of this drug after the introduction of a tamper‐deterrent formulation in February 2012. We observed no obvious “rebound” in national oxycodone dispensing after the introduction of generic non‐tamper‐deterrent products later in the same year. However, we did observe considerable variation in the market shares achieved by the generic forms across the Canadian provinces. The uptake was highest in Quebec, followed by British Columbia, Ontario, and Newfoundland and Labrador.

The observed reduction in dispensing of controlled‐release oxycodone at the time of the introduction of the tamper‐deterrent formulation is aligned with changes in its listing on the majority of provincial drug insurance plans in Canada, which either chose not to list this new form of oxycodone on their formularies or listed it with exceptional access programs that required patients to meet a number of strict eligibility criteria before accessing the drug. Alberta, the only province to list OxyNeo as a full benefit on their formulary, had only minimal change in their dispensing patterns for controlled‐release oxycodone in February 2012 and was the province with the highest rate of prescribing by the end of our study period. This suggests that public drug funding and restricted reimbursement criteria influenced broad patterns of oxycodone use across Canada. However, these products have a relatively low cost, which might not deter their use in cases where a patient is prescribed the product by their physician but do not meet coverage criteria by a public or private drug insurance plan. Our study also suggests that the listing status of generic controlled‐release oxycodone had a significant impact on the uptake of these products. This is most apparent in Quebec where both brand (OxyNeo) and generic controlled‐release oxycodone are listed similarly on the drug formulary, with interchangeability and application of lowest prices in effect. This listing creates a financial incentive for the generic form of this drug to be dispensed, which may have led to the high degree of controlled‐release oxycodone dispensed in the province being for this non‐tamper‐deterrent form. Similarly, in British Columbia, the provincial drug formulary reimbursed the generic form until February 2015 when it was announced that the PharmaCare program would no longer cover these products.^18^ Despite this announcement, there was no change in the pattern of generic controlled‐release oxycodone in the province over the subsequent 14 months. This suggests that reimbursement by the provincial drug program was not the only driver of this higher use of generic controlled‐release oxycodone in British Columbia and that patients continued to access these products through private insurance coverage or cash purchase.

In Ontario and Newfoundland and Labrador, there was a relatively high uptake of generic controlled‐release oxycodone despite these products not being listed on the provincial drug formularies. In Ontario, where oxycodone has historically been the dominant long‐acting opioid on the market,^1^, ^16^ it is possible that people who could no longer access this product through the provincial drug formulary once restrictions were implemented around OxyNeo reimbursement, chose to pay out of pocket for the cheaper, generic form of this drug, instead of being switched to another long‐acting opioid product. Furthermore, it is possible that there was a higher degree of OxyContin misuse (eg, crushing and chewing tablets) in these provinces,^19^, ^20^ which could have led to increased desirability of the generic non‐tamper‐deterrent form of oxycodone.

### 5.1 Strengths and limitations

The core strength of this study is our ability to report changes in the dispensing patterns of all controlled‐release oxycodone prescriptions dispensed over more than 8 years across Canada at the national and provincial level. However, several limitations warrant emphasis. First, we did not have access to patient‐level data and, therefore, could not determine the degree to which generic controlled‐release oxycodone tablets were clustered at the individual level or rates of initiation of these products. Second, the Compuscript database only captures prescriptions dispensed from community pharmacies and, therefore, cannot comment on patterns of hospital use of controlled‐release oxycodone. Finally, we are not able to distinguish between publicly and privately funded (ie, cash paid or privately insured) prescriptions. However, given that generic forms of controlled‐release oxycodone are generally not covered by public drug programs, this should have limited impact on our interpretation of these results.

## 6 CONCLUSIONS

Changes to the availability and listing status of generic and brand name formulations of controlled‐release oxycodone have led to important changes in both the rate of use of these products across Canada and the relative market share of non‐tamper‐deterrent opioids. Importantly, although the introduction of a tamper‐deterrent formulation in early 2012 led to considerable reductions in the rate of controlled‐release oxycodone dispensing, the marketing of generic non‐tamper‐deterrent formulations later that year did not lead to any subsequent expansion of the market. By 2016, 1 in 5 tablets for controlled‐release oxycodone that were dispensed in Canada were for a generic, non‐tamper‐deterrent form, and in several provinces, this was likely paid for by private insurers or cash payments. The geographic variation in uptake of these products suggests that provincial drug reimbursement policies—including restrictions on access through public drug formularies—has influenced these trends. In Canada, there was sufficient uptake of generic non‐tamper‐deterrent formulations of oxycodone to justify further research to determine if this has led to measurable changes in the safety of oxycodone use in Canada. This is important as drug regulators from around the world consider the role of tamper‐deterrent opioid formulations in addressing the opioid crisis and seek to understand how patent expiration and generic non‐tamper‐deterrent alternatives will impact the prescribed opioid environment.

## CONFLICT OF INTEREST

Tara Gomes has received unrestricted grant funding from the Ontario Ministry of Health and Long‐Term Care. No other authors report any competing interests.

## Supporting information

Appendix S1: Controlled Release Oxycodone Coverage Through Provincial Public Drug Programs
