# Supplementary material for: Trends and uptake of new formulations of controlled‐release oxycodone in Canada
Source: Pharmacoepidemiol Drug Saf. 2018 Jan 23;27(5):520–5. doi: 10.1002/pds.4390 (PMC5947657; doi:10.1002/pds.4390)
Supplement: Supplementary file 1 — Appendix S1: Controlled Release Oxycodone Coverage Through Provincial Public Drug Programs [file PDS-27-520-s001.docx]

**Supplementary Appendix: Controlled Release Oxycodone Coverage Through Provincial Public Drug Programs**

| **Province** | **Listing of OxyNeo** | **Listing of Generics** |
| --- | --- | --- |
| **BC** | Listed for patients covered by the Palliative Care Drug Plan and for other patients on an exceptional basis through PharmaCare’s Special Authority process; | Initially reimbursed. February 2015: generic controlled-release oxycodone no longer covered |
| **Alberta** | Listed as a regular benefit | Not listed |
| **Saskatchewan** | Listed under the Exception Drug Status (EDS) program for the treatment of pain in palliative and cancer patients. | Not listed |
| **Manitoba** | Listed under the Exception Drug Status for the diagnosis of cancer related pain or other chronic pain diagnoses where the patient is unable to tolerate or receive an adequate response to either the regular release dosage forms of oxycodone or the sustained release preparations of morphine or hydromorphone. | Not listed |
| **Ontario** | Listed for palliative care patients, and under the Exceptional Access Program for the treatment of chronic pain in patients who have experienced intolerance or have failed an adequate trial of at least one other listed long-acting opioid product. OxyNeo 60mg and 80mg are not funded. | Not listed |
| **Quebec** | Listed on the Exceptional Access list when two other opioids are not tolerated, contraindicated or ineffective. | Listed similarly to OxyNeo. The patient must pay the difference between OxyNeo and the generic if they want to access OxyNeo. |
| **New Brunswick** | Not listed, but reimbursed for patients who were taking OxyContin when it was discontinued. | Not listed |
| **Nova Scotia** | Not listed, but reimbursed for patients who were taking OxyContin when it was discontinued. Accessible for palliative care patients on a case by case basis. | Listed similarly to OxyNeo |
| **Newfoundland & Labrador** | Not listed, but it is reimbursed for patients who were taking OxyContin when it was discontinued. | Not listed |
| **PEI** | Not listed, but reimbursed for patients who were taking OxyContin in the 90 days prior to it being discontinued. | Not listed |
